# Supplementary material for: Effective coverage for maternal health: operationalising effective coverage cascades for antenatal care and nutrition interventions for pregnant women in seven low- and middle-income countries
Source: J Glob Health. 2025 Apr 11;15:04041. doi: 10.7189/jogh.15.04041 (PMC11987578; doi:10.7189/jogh.15.04041)

Supplement to: Sheffel A, Carter E, Heidkamp R, Hossain A, Katz J, Kim S, Lama T, Marchant T, Perin J, Requejo J, Walton S, Munos M. Effective coverage for maternal health: operationalising effective coverage cascades for antenatal care and nutrition interventions for pregnant women in seven low- and middle-income countries. J Glob Health. 2025;15:04041.

Table S1: Household survey data sources

Country	HHS	Study design details	Sample size
Bangladesh	DHS 2014	Sample representative nationally as well as of urban and rural areas and division	3,217 women with a birth in the last two years
Haiti	DHS 2016-17	Sample representative nationally as well as of urban and rural areas and department	2,583 women with a birth in the last two years
Malawi	DHS 2015-16	Sample representative nationally as well as of urban and rural areas, region, and district	6,814 women with a birth in the last two years
Nepal	DHS 2016	Sample representative nationally as well as of urban and rural areas, development region, and province	2,007 women with a birth in the last two years
Senegal	DHS 2017	Sample representative nationally as well as of urban and rural areas and region	4,926 women with a birth in the last two years
Sierra Leone	DHS 2019	Sample representative nationally as well as of urban and rural areas, region, and district	4,057 women with a birth in the last two years
Tanzania	DHS 2015-16	Sample representative nationally as well as of urban and rural areas, zone, and region (for most indicators)	4,283 women with a birth in the last two years

Table S2: Health facility assessment data sources

Country	HFA	Census or Sample	Study design details	Modules implemented
Bangladesh	SPA 2014	Sample	Stratified random sample of 1,596 health facilities selected from all formal-sector health facilities in Bangladesh. Designed to be representative of the seven administrative divisions and by facility type.	Facility inventory
Haiti	SPA 2013	Census	Census of all health facilities (public and private) with a sample of providers and clients at facilities.	Facility inventory, ANC client observation, ANC client exit interview
Malawi	SPA 2013-14	Census	Census of all health facilities (public and private) with a sample of providers and clients at facilities.	Facility inventory, ANC client observation, ANC client exit interview
Nepal	SPA 2015	Sample	Stratified random sample of 992 health facilities selected from all formal-sector health facilities in Nepal. Designed to be representative of the three geo-ecological regions in the country, the 13 development-ecological zones, by facility type, and by managing authority.	Facility inventory, ANC client observation, ANC client exit interview
Senegal	SPA 2016	Sample	Stratified random sample of 484 health facilities selected from all formal-sector health facilities in Senegal. Designed to be representative by facility type.	Facility inventory, ANC client observation, ANC client exit interview
Sierra Leone	SARA 2019	Census	Census of all health facilities (public and private).	Facility inventory
Tanzania	SPA 2014-15	Sample	Stratified random sample of 1,200 health facilities selected from all formal-sector health facilities in Tanzania. Designed to be representative by region, facility type, and managing authority.	Facility inventory, ANC client observation, ANC client exit interview

Table S3: ANC readiness and provision/experience of care items and detailed definitions

No	Domain	Code	Item	Detailed indicator definition	Data availability notes	Survey specific: Indicator not collected	Cross-country differences
**Readiness**							
1	Equipment and supplies	ANC_READY_03	Blood pressure apparatus	Either manual or digital blood pressure apparatus observed available and functional in the ANC service provision area.	SARA only asks about this item in the OPD		Sierra Leone is OPD instead of ANC service area
2	Equipment and supplies	ANC_READY_30	Stethoscope	Stethoscope observed available and functional in the ANC service provision area.	SARA only asks about this item in the OPD		Sierra Leone is OPD instead of ANC service area
3	Equipment and supplies	ANC_READY_22	Examination bed	Examination bed observed available in the ANC service provision area.	Not collected in SARA	SLE 2017	
4	Equipment and supplies	GEN_READY_17	Latex gloves	Clean/sterile disposable latex gloves observed available in the OPD or ANC service provision area.	SARA only asks about this item in the OPD		Sierra Leone is OPD only
5	Equipment and supplies	GEN_READY_15	Auto-disable syringes with needles; single use standard disposable syringes with needles	Single-use standard disposable syringes with needles or auto-disable syringes with needles observed available anywhere in the facility.	SARA only asks about this item in the OPD whereas SPA is in multiple service areas		Sierra Leone is OPD only
6	Equipment and supplies	GEN_READY_16	Handwashing materials	Either handwashing soap and running water OR alcohol-based hand sanitizer observed available in the OPD or ANC service provision area.	SARA only asks about this item in the OPD		Sierra Leone is OPD only
7	Equipment and supplies	GEN_READY_14	Environmental disinfectant	Disinfectant (e.g., chlorine, hibitane, alcohol) observed available anywhere in the facility.	SARA only asks about this item in the OPD whereas SPA is in multiple service areas		Sierra Leone is OPD only
8	Diagnostics	ANC_READY_04	Hemoglobin	Facility has the ability to conduct hemoglobin testing onsite and has the required equipment available and functioning/unexpired (rapid test OR hematology analyzer OR hemocue and microcuvette OR colorimeter or hemoglobinometer & drabkin's solution & pipette OR litmus paper for hemoglobin test).			Haiti, Malawi, Nepal, Senegal, and Bangladesh don't include functioning of microcuvette or drabkin solution unexpired<br>Nepal also doesn't include litmus paper for hemoglobin test<br>Sierra Leone only includes heomcue, colorimeter, hemoglobinometer

No	Domain	Code	Item	Detailed indicator definition	Data availability notes	Survey specific: Indicator not collected	Cross-country differences
9	Diagnostics	ANC_READY_05	Urine dipstick- protein	Facility has the ability to conduct urine protein dipstick testing onsite and has the required equipment available and functioning/unexpired (dipsticks for urine protein)			
10	Diagnostics	ANC_READY_23	Urine dipstick- glucose	Facility has the ability to conduct urine glucose dipstick testing onsite and has the required equipment available and functioning/unexpired (dipsticks for urine glucose)			
11	Diagnostics	ANC_READY_25	Syphilis RDT / RPR	Facility has the ability to conduct syphilis RDT / RPR testing onsite and has the required equipment available and functioning/unexpired (RDT, PCR, VDLR, RPR, rotator/shaker).			Sierra Leone doesn't ask about rotator/shaker
12	Diagnostics	ANC_READY_26	HIV diagnostic capacity	Facility has the ability to conduct HIV testing onsite and has the required equipment available and functioning/unexpired (RDT, ELISA (scanner/reader, washer, assay, and incubator), dynabeads, or Western blot).		BGD 2014	BGD does not assess HIV<br>MWI doesn't ask about expiration of western blot or availability of ELISA assay or incubator<br>MWI doesn't ask about expiration of western blot or availability of ELISA assay<br>NPL doesn't ask about expiration of western blot, RDT in ANC service site, RDT in FP service site<br>Sierra Leone doesn't ask about dynabeads or Western blot, RDT is only asked in one location
13	Medicines and commodities	ANC_READY_06	Iron tablets	Iron tablets or iron + folic acid combination tablets observed in pharmacy or anywhere in the facility where medicines are routinely stored; at least one with valid expiration date.			
14	Medicines and commodities	ANC_READY_07	Folic acid tablets	Folic acid tablets or iron + folic acid combination tablets observed in pharmacy or anywhere in the facility where medicines are routinely stored; at least one with valid expiration date.			

No	Domain	Code	Item	Detailed indicator definition	Data availability notes	Survey specific: Indicator not collected	Cross-country differences
15	Medicines and commodities	ANC_READY_08	Tetanus toxoid vaccine	Tetanus toxoid vaccine observed in pharmacy or anywhere in the facility where medicines are routinely stored; at least one with valid expiration date.			
16	Basic amenities	GEN_READY_02	Improved water source	Facility has an improved water source that is onsite or within 500meters of the facility.			
17	Basic amenities	GEN_READY_03	Room with auditory and visual privacy	Room is private room with auditory and visual privacy is observed in the OPD.			
18	Basic amenities	GEN_READY_04	Sanitation facilities	Facility has a functioning toilet (latrine) available for general outpatient client use.			
19	Human resources	ANC_READY_02	Staff trained in the past 2 years in antenatal care (broad ANC training)	Proportion of health care workers delivering ANC services that have been trained in antenatal care (regardless of the specific topic area) in the last two years.<br><br>Alternative: At least one health care worker providing ANC services has been trained in antenatal care (regardless of the specific topic area) in the last two years.			Sierra Leone uses alternative definition while SPA countries use preferred/ original definition
**Provision/experience of care**							
20	History taking	ANC_PROV_01	Provider asked about client age	During the observed ANC visit, the provider took a personal history and asked about the client's age.	Not collected in SARA; Collected only in some SPAs (those that include client/provider observation)	BGD 2014 SPA<br>SLE 2017 SARA	
21	History taking	ANC_PROV_02	Provider asked about medications client is taking	During the observed ANC visit, the provider took a personal history and asked about medications the client is taking.	Not collected in SARA; Collected only in some SPAs (those that include client/provider observation)	BGD 2014 SPA<br>SLE 2017 SARA	
22	History taking	ANC_PROV_03	Provider asked about date last menstrual period began	During the observed ANC visit, the provider took a personal history and asked about the date the client's last menstrual period began.	Not collected in SARA; Collected only in some SPAs (those that include client/provider observation)	BGD 2014 SPA<br>SLE 2017 SARA	

No	Domain	Code	Item	Detailed indicator definition	Data availability notes	Survey specific: Indicator not collected	Cross-country differences
23	History taking	ANC_PROV_04	Provider asked about any prior pregnancy	During the observed ANC visit, the provider took a personal history and asked about any prior pregnancy.	Not collected in SARA; Collected only in some SPAs (those that include client/provider observation)	BGD 2014 SPA<br>SLE 2017 SARA	
24	History taking	ANC_PROV_06	Provider asked about previous still birth	During the observed ANC visit, the provider took a medical history for prior pregnancies and asked about still birth.	Not collected in SARA; Collected only in some SPAs (those that include client/provider observation)	BGD 2014 SPA<br>SLE 2017 SARA	
25	History taking	ANC_PROV_07	Provider asked about prior pregnancy in which the infant died in the first week of life	During the observed ANC visit, the provider took a medical history for prior pregnancies and asked about any infant that died in the first week of life.	Not collected in SARA; Collected only in some SPAs (those that include client/provider observation)	BGD 2014 SPA<br>SLE 2017 SARA	
26	History taking	ANC_PROV_08	Provider asked about heavy bleeding during or after delivery in prior pregnancies	During the observed ANC visit, the provider took a medical history for prior pregnancies and asked about heavy bleeding during or after delivery.	Not collected in SARA; Collected only in some SPAs (those that include client/provider observation)	BGD 2014 SPA<br>SLE 2017 SARA	
27	History taking	ANC_PROV_09	Provider asked about previous assisted delivery	During the observed ANC visit, the provider took a medical history for prior pregnancies and asked about previous assisted delivery.	Not collected in SARA; Collected only in some SPAs (those that include client/provider observation)	BGD 2014 SPA<br>SLE 2017 SARA	
28	History taking	ANC_PROV_10	Provider asked about previous spontaneous abortion	During the observed ANC visit, the provider took a medical history for prior pregnancies and asked about previous spontaneous abortion.	Not collected in SARA; Collected only in some SPAs (those that include client/provider observation)	BGD 2014 SPA<br>SLE 2017 SARA	
29	History taking	ANC_PROV_11	Provider asked about previous multiple pregnancies	During the observed ANC visit, the provider took a medical history for prior pregnancies and asked about previous multiple pregnancies	Not collected in SARA; Collected only in some SPAs (those that include client/provider observation)	BGD 2014 SPA<br>SLE 2017 SARA	

No	Domain	Code	Item	Detailed indicator definition	Data availability notes	Survey specific: Indicator not collected	Cross-country differences
30	History taking	ANC_PROV_12	Provider asked about previous prolonged labor	During the observed ANC visit, the provider took a medical history for prior pregnancies and asked about previous prolonged labor.	Not collected in SARA; Collected only in some SPAs (those that include client/provider observation)	BGD 2014 SPA<br>SLE 2017 SARA	
31	History taking	ANC_PROV_13	Provider asked about previous pregnancy-induced hypertension	During the observed ANC visit, the provider took a medical history for prior pregnancies and asked about pregnancy-induced hypertension.	Not collected in SARA; Collected only in some SPAs (those that include client/provider observation)	BGD 2014 SPA<br>SLE 2017 SARA	
32	History taking	ANC_PROV_14	Provider asked about previous pregnancy related convulsions	During the observed ANC visit, the provider took a medical history for prior pregnancies and asked about pregnancy related convulsions.	Not collected in SARA; Collected only in some SPAs (those that include client/provider observation)	BGD 2014 SPA<br>SLE 2017 SARA	
33	History taking	ANC_PROV_15	Provider asked about high fever or infection during prior pregnancy/pregnancies	During the observed ANC visit, the provider took a medical history for prior pregnancies and asked about high fever or infection during a prior pregnancy/pregnancies.	Not collected in SARA; Collected only in some SPAs (those that include client/provider observation)	BGD 2014 SPA<br>SLE 2017 SARA	
34	History taking	ANC_PROV_17	Provider asked about vaginal bleeding in current pregnancy	During the observed ANC visit, the provider took a history of complaints in the current pregnancy and asked about vaginal bleeding.	Not collected in SARA; Collected only in some SPAs (those that include client/provider observation)	BGD 2014 SPA<br>SLE 2017 SARA	
35	History taking	ANC_PROV_18	Provider asked about fever in current pregnancy	During the observed ANC visit, the provider took a history of complaints in the current pregnancy and asked about fever.	Not collected in SARA; Collected only in some SPAs (those that include client/provider observation)	BGD 2014 SPA<br>SLE 2017 SARA	
36	History taking	ANC_PROV_19	Provider asked about headache or blurred vision in current pregnancy	During the observed ANC visit, the provider took a history of complaints in the current pregnancy and asked about headache or blurred vision.	Not collected in SARA; Collected only in some SPAs (those that include client/provider observation)	BGD 2014 SPA<br>SLE 2017 SARA	

No	Domain	Code	Item	Detailed indicator definition	Data availability notes	Survey specific: Indicator not collected	Cross-country differences
37	History taking	ANC_PROV_20	Provider asked about swollen face or hands or extremities in current pregnancy	During the observed ANC visit, the provider took a history of complaints in the current pregnancy and asked about swollen face or hands or extremities.	Not collected in SARA; Collected only in some SPAs (those that include client/provider observation)	BGD 2014 SPA<br>SLE 2017 SARA	
38	History taking	ANC_PROV_21	Provider asked about tiredness or breathlessness in current pregnancy	During the observed ANC visit, the provider took a history of complaints in the current pregnancy and asked about tiredness or breathlessness.	Not collected in SARA; Collected only in some SPAs (those that include client/provider observation)	BGD 2014 SPA<br>SLE 2017 SARA	
39	History taking	ANC_PROV_22	Provider asked about fetal movement (loss of, excessive, normal) in current pregnancy	During the observed ANC visit, the provider took a history of complaints in the current pregnancy and asked about fetal movement (loss of, excessive, normal).	Not collected in SARA; Collected only in some SPAs (those that include client/provider observation)	BGD 2014 SPA<br>SLE 2017 SARA	
40	History taking	ANC_PROV_23	Provider asked about cough or difficulty breathing for 3 weeks or longer in current pregnancy	During the observed ANC visit, the provider took a history of complaints in the current pregnancy and asked about cough or difficulty breathing for 3 weeks or longer.	Not collected in SARA; Collected only in some SPAs (those that include client/provider observation)	BGD 2014 SPA<br>SLE 2017 SARA	
41	Examination and observation	ANC_PROV_28	Observed client for oedema	During the observed ANC visit, the provider examined the client including observing the client for oedema (other than ankle-specific).	Not collected in SARA; Collected only in some SPAs (those that include client/provider observation)	BGD 2014 SPA<br>SLE 2017 SARA	
42	Examination and observation	ANC_PROV_30	Examined client blood pressure	During the observed ANC visit, the provider examined the client including taking a blood pressure measurement.	Not collected in SARA; Collected only in some SPAs (those that include client/provider observation)	BGD 2014 SPA<br>SLE 2017 SARA	
43	Examination and observation	ANC_PROV_31	Examined client weight	During the observed ANC visit, the provider examined the client including taking a weight measurement.	Not collected in SARA; Collected only in some SPAs (those that include client/provider observation)	BGD 2014 SPA<br>SLE 2017 SARA	

No	Domain	Code	Item	Detailed indicator definition	Data availability notes	Survey specific: Indicator not collected	Cross-country differences
44	Examination and observation	ANC_PROV_33	Palpated the clients' abdomen for fundal height	During the observed ANC visit, the provider examined the client including palpating the clients abdomen for fundal height.	Not collected in SARA; Collected only in some SPAs (those that include client/provider observation)	BGD 2014 SPA<br>SLE 2017 SARA	
45	Laboratory investigations	ANC_PROV_37	Performed or referred the client for hemoglobin testing	During the observed ANC visit, the provider performed or referred the client for hemoglobin testing.	Not collected in SARA; Collected only in some SPAs (those that include client/provider observation)	BGD 2014 SPA<br>SLE 2017 SARA	
46	Laboratory investigations	ANC_PROV_38	Performed or referred the client for grouping and rhesus factor testing	During the observed ANC visit, the provider performed or referred the client for grouping and rhesus factor testing.	Not collected in SARA; Collected only in some SPAs (those that include client/provider observation)	BGD 2014 SPA<br>SLE 2017 SARA	
47	Laboratory investigations	ANC_PROV_39	Performed or referred the client for syphilis testing	During the observed ANC visit, the provider performed or referred the client for syphilis testing.	Not collected in SARA; Collected only in some SPAs (those that include client/provider observation)	BGD 2014 SPA<br>SLE 2017 SARA	
48	Laboratory investigations	ANC_PROV_40	Performed or referred the client for HIV testing	During the observed ANC visit, the provider performed or referred the client for HIV testing.	Not collected in SARA; Collected only in some SPAs (those that include client/provider observation)	BGD 2014 SPA<br>SLE 2017 SARA	
49	Laboratory investigations	ANC_PROV_41	Performed or referred the client for urine-protein, sugar, acetone testing	During the observed ANC visit, the provider performed or referred the client for urine protein, sugar, and acetone testing	Not collected in SARA; Collected only in some SPAs (those that include client/provider observation)	BGD 2014 SPA<br>SLE 2017 SARA	
50	Preventative treatment	ANC_PROV_43	Provided or prescribed iron pills and/or folic acid pills	During the observed ANC visit, the provider prescribed or provided iron pills or folic acid or both	Not collected in SARA; Collected only in some SPAs (those that include client/provider observation)	BGD 2014 SPA<br>SLE 2017 SARA	

No	Domain	Code	Item	Detailed indicator definition	Data availability notes	Survey specific: Indicator not collected	Cross-country differences
51	Preventative treatment	ANC_PROV_45	Provided or prescribed tetanus toxoid immunization	During the observed ANC visit, the provider administered or prescribed tetanus toxoid immunization	Not collected in SARA; Collected only in some SPAs (those that include client/provider observation)	BGD 2014 SPA<br>SLE 2017 SARA	
52	Client education and counseling	ANC_PROV_47	Discussed process of pregnancy and its complications	During the observed ANC visit, the provider discussed the process of pregnancy and its complications	Not collected in SARA; Collected only in some SPAs (those that include client/provider observation)	BGD 2014 SPA<br>SLE 2017 SARA	
53	Client education and counseling	ANC_PROV_48	Discussed diet and nutrition	During the observed ANC visit, the provider discussed diet and nutrition (i.e., quantity and quality of food to eat) during the pregnancy.	Not collected in SARA; Collected only in some SPAs (those that include client/provider observation)	BGD 2014 SPA<br>SLE 2017 SARA	
54	Client education and counseling	ANC_PROV_58	Discussed danger signs in pregnancy	During the observed ANC visit, the provider discussed danger signs in pregnancy.	Not collected in SARA; Collected only in some SPAs (those that include client/provider observation)	BGD 2014 SPA<br>SLE 2017 SARA	
55	Client education and counseling	ANC_PROV_49	Discussed voluntary counselling and testing for HIV	During the observed ANC visit, the provider discussed voluntary counselling and testing for HIV	Not collected in SARA; Collected only in some SPAs (those that include client/provider observation)	BGD 2014 SPA<br>SLE 2017 SARA	
56	Client education and counseling	ANC_PROV_50	Discussed breastfeeding	During the observed ANC visit, the provider discussed early initiation and prolonged breastfeeding as well as exclusive breastfeeding.	Not collected in SARA; Collected only in some SPAs (those that include client/provider observation)	BGD 2014 SPA<br>SLE 2017 SARA	
57	Client education and counseling	ANC_PROV_51	Discussed plans of delivery	During the observed ANC visit, the provider discussed plans of delivery (i.e., emergency preparedness, place of delivery, transportation, financial arrangements).	Not collected in SARA; Collected only in some SPAs (those that include client/provider observation)	BGD 2014 SPA<br>SLE 2017 SARA	

No	Domain	Code	Item	Detailed indicator definition	Data availability notes	Survey specific: Indicator not collected	Cross-country differences
58	Client experience	ANC_SATIS_02a	Client satisfied with ability to discuss problems or concerns about pregnancy with provider	Client had no problems with regards to her ability to discuss the problems or concerns about her pregnancy.	Not collected in SARA; Collected only in some SPAs (those that include client/provider observation)	BGD 2014 SPA<br>SLE 2017 SARA	
59	Client experience	ANC_SATIS_03a	Client satisfied with the amount of explanation received about the problem or treatment	Client had no problems with regards to the amount of explanation she received about the problem or treatment.	Not collected in SARA; Collected only in some SPAs (those that include client/provider observation)	BGD 2014 SPA<br>SLE 2017 SARA	
60	Client experience	ANC_SATIS_10a	Client satisfied with how treated by the staff	Client had no problems with regards to how the staff treated her.	Not collected in SARA; Collected only in some SPAs (those that include client/provider observation)	BGD 2014 SPA<br>SLE 2017 SARA	
61	Client experience	ANC_SATIS_04a	Client satisfied with privacy from having others see	Client had no problems with regards to privacy from having others see.	Not collected in SARA; Collected only in some SPAs (those that include client/provider observation)	BGD 2014 SPA<br>SLE 2017 SARA	
62	Client experience	ANC_SATIS_05a	Client satisfied with privacy from having others hear	Client had no problems with regards to privacy from having others hear.	Not collected in SARA; Collected only in some SPAs (those that include client/provider observation)	BGD 2014 SPA<br>SLE 2017 SARA	
63	Client experience	ANC_SATIS_01a	Client satisfied with time waited	Client had no problems with regards to the time she waited.	Not collected in SARA; Collected only in some SPAs (those that include client/provider observation)	BGD 2014 SPA<br>SLE 2017 SARA	
64	Client experience	ANC_SATIS_08a	Client satisfied with number of days services are available	Client had no problems with regards to the number of days services are available.	Not collected in SARA; Collected only in some SPAs (those that include client/provider observation)	BGD 2014 SPA<br>SLE 2017 SARA	

No	Domain	Code	Item	Detailed indicator definition	Data availability notes	Survey specific: Indicator not collected	Cross-country differences
65	Client experience	ANC_SATIS_07a	Client satisfied with the hours of service	Client had no problems with regards to the hours of service	Not collected in SARA; Collected only in some SPAs (those that include client/provider observation)	BGD 2014 SPA<br>SLE 2017 SARA	
66	Client experience	ANC_SATIS_11a	Client satisfied with the cost for services or treatments	Client had no problems with regards to the cost for services or treatment	Not collected in SARA; Collected only in some SPAs (those that include client/provider observation)	BGD 2014 SPA<br>SLE 2017 SARA	
67	Client experience	ANC_SATIS_06a	Client satisfied with the availability of medicines	Client had no problems with regards to the availability of medicines	Not collected in SARA; Collected only in some SPAs (those that include client/provider observation)	BGD 2014 SPA<br>SLE 2017 SARA	
68	Client experience	ANC_SATIS_09a	Client satisfied with the cleanliness of the facility	Client had no problems with regards to the cleanliness of the facility	Not collected in SARA; Collected only in some SPAs (those that include client/provider observation)	BGD 2014 SPA<br>SLE 2017 SARA	

Table S4: Maternal nutrition readiness and provision/experience of care items and detailed definitions

No	Domain	Code	Item	Detailed indicator definition	Data availability notes	Survey specific: Indicator not collected	Cross-country differences
**Readiness**							
1	Equipment and supplies	ANC_READY_21	Adult weighing scale	Adult weighing scale observed available and functional in the OPD or ANC service provision area.	SARA only asks about this item in the OPD		Sierra Leone is OPD only
2	Equipment and supplies	ANC_READY_29	Stadiometer or height rod	Stadiometer or height rod/height board observed available and functional in the OPD or ANC service provision area.	Collected only in some SPAs<br>SARA only asks about this item in the OPD	BGD 2014<br>HTI 2013<br>NPL 2015<br>SEN 2016	Sierra Leone is OPD only
3	Equipment and supplies	GEN_READY_17	Latex gloves	Clean/sterile disposable latex gloves observed available in the OPD or ANC service provision area.	SARA only asks about this item in the OPD		Sierra Leone is OPD only
4	Equipment and supplies	GEN_READY_16	Handwashing materials	Either handwashing soap and running water OR alcohol-based hand sanitizer observed available in the OPD or ANC service provision area.	SARA only asks about this item in the OPD		Sierra Leone is OPD only
5	Equipment and supplies	GEN_READY_14	Environmental disinfectant	Disinfectant (e.g., chlorine, hibitane, alcohol) observed available anywhere in the facility.	SARA only asks about this item in the OPD whereas SPA is in multiple service areas		Sierra Leone is OPD only
6	Equipment and supplies	GEN_READY_15	Auto-disable syringes with needles; single use standard disposable syringes with needles	Single-use standard disposable syringes with needles or auto-disable syringes with needles observed available anywhere in the facility.	SARA only asks about this item in the OPD whereas SPA is in multiple service areas		Sierra Leone is OPD only
7	Diagnostics	ANC_READY_41	Blood glucose	Facility has the ability to conduct blood glucose testing onsite and has the required equipment available and functioning/unexpired (glucometer and test strips OR blood chemistry analyzer)			
8	Diagnostics	ANC_READY_04	Hemoglobin	Facility has the ability to conduct hemoglobin testing onsite and has the required equipment available and functioning/unexpired (rapid test OR hematology analyzer OR hemocue and microcuvette OR colorimeter or hemoglobinometer & drabkin's solution & pipette OR litmus paper for hemoglobin test).			Haiti, Malawi, Nepal, Senegal, and Bangladesh don't include functioning of microcuvette or drabkin solution unexpired<br>Nepal also doesn't include litmus paper for hemoglobin test<br>Sierra Leone only

							includes heomcue, colorimeter, hemoglobinometer
9	Medicines and commodities	ANC_READY_31	Albendazole/ mebendazole	Albendazole or mebendazole tablets for deworming observed in pharmacy or anywhere in the facility where medicines are routinely stored; at least one with valid expiration date.			
10	Medicines and commodities	ANC_READY_07	Folic acid tablets	Folic acid tablets or iron + folic acid combination tablets observed in pharmacy or anywhere in the facility where medicines are routinely stored; at least one with valid expiration date.			
11	Medicines and commodities	ANC_READY_06	Iron tablets	Iron tablets (stand-alone, not in combination with folic acid) observed in pharmacy or anywhere in the facility where medicines are routinely stored; at least one with valid expiration date.			
12	Medicines and commodities	ANC_READY_09	Sulfadoxine-pyrimethamine (SP) tablets	Sulfadoxine-pyrimethamine (SP) tablets for IPTp observed in pharmacy or anywhere in the facility where medicines are routinely stored; at least one with valid expiration date.	Collected only in some SPAs; malaria endemic countries	BGD 2014<br>HTI 2013<br>NPL 2015	
13	Human resources	ANC_READY_01	Guidelines for antenatal care	Guidelines for antenatal care are observed available in the antenatal care service provision area.			
14	Human resources	ANC_READY_44	Guidelines for intermittent preventive treatment of malaria during pregnancy	Guidelines for intermittent preventive treatment of malaria during pregnancy are observed available in the antenatal care service provision area.	Collected only in some SPAs; malaria endemic countries	BGD 2014<br>HTI 2013<br>NPL 2015	
15	Human resources	ANC_READY_43	Guidelines for infant and young child feeding	Guidelines for infant and young child feeding are observed available in the PMTCT service provision area.	Collected only in some SPAs; high HIV burden countries	BGD 2014<br>NPL 2015	
16	Human resources	ANC_READY_02	Staff trained in the past 2 years in antenatal care (broad ANC training)	Proportion of health care workers delivering ANC services that have been trained in antenatal care (regardless of the specific topic area) in the last two years.<br><br>Alternative: At least one health care worker providing ANC services has been trained in antenatal care (regardless of the specific topic area) in the last two years.			Sierra Leone uses alternative definition while SPA countries use preferred/ original definition
17	Human resources	ANC_READY_45	Staff trained in the past 2 years in ANC	Proportion of health care workers delivering ANC services that have been	Not collected in SARA	SLE 2017	

			screening (e.g., blood pressure, urine glucose, and protein)	trained in ANC screening (e.g., blood pressure, urine glucose, and protein) in the last two years.			
18	Human resources	ANC_READY_46	Staff trained in the past 2 years in counseling for ANC (e.g., nutrition, FP, and newborn care)	Proportion of health care workers delivering ANC services that have been trained in ANC screening (e.g., blood pressure, urine glucose, and protein) in the last two years.	Not collected in SARA	SLE 2017	
19	Human resources	ANC_READY_33	Staff trained in the past 2 years in nutritional assessment of the pregnant woman (e.g., BMI calculation, MUAC measurement)	Proportion of health care workers delivering ANC services that have been trained in nutritional assessment of the pregnant woman (e.g., BMI calculation, MUAC measurement) in the last two years.	Not collected in SARA	SLE 2017	
20	Human resources	ANC_READY_47	Staff trained in the past 2 years in complications of pregnancy and their management	Proportion of health care workers delivering ANC services that have been trained in complications of pregnancy and their management in the last two years.	Not collected in SARA	SLE 2017	
21	Human resources	ANC_READY_48	Staff trained in the past 2 years in infant and young child feeding	Proportion of health care workers delivering ANC services that have been trained in infant and young child feeding in the last two years.			Sierra Leone uses alternative definition while SPA countries use preferred/ original definition
22	Basic amenities	GEN_READY_40	Clean environment	General assessment of cleanliness/conditions of the facility; score out of 8 possible good responses including the following eight areas: Floor swept, no obvious dirt, Counters/tables/chairs wiped, Needles/sharps outside of sharps container, Sharps box overflowing/torn/pierced, Bandages/ infectious waste lying uncovered, Walls with significant damage, Doors with significant damage, Ceiling with water stains/ damage	Not collected in SARA	SLE 2017	
23	Basic amenities	GEN_READY_07	Emergency transportation	Facility has a functioning vehicle with fuel that is routinely available that can be used for emergency transportation or access to a vehicle in near proximity that can be used for emergency transportation			
24	Basic amenities	GEN_READY_02	Improved water source	Facility has an improved water source that is onsite or within 500meters of the facility.			

25	Basic amenities	ANC_READY_22	Place for women to sit/lie down	Examination bed observed available in the ANC service provision area.	Not collected in SARA	SLE 2017	
26	Basic amenities	GEN_READY_01	Power	Facility is connected to the central supply electricity grid and electricity is always available, or facility has a functional backup generator with fuel (or charged battery), or facility has access to solar power			
27	Basic amenities	GEN_READY_04	Sanitation facilities	Facility has a functioning toilet (latrine) available for general outpatient client use.			
**Provision/experience of care**							
28	Laboratory investigations	ANC_PROV_37	Performed or referred the client for hemoglobin testing	During the observed ANC visit, the provider performed or referred the client for hemoglobin testing.	Not collected in SARA; Collected only in some SPAs (those that include client/provider observation)	BGD 2014<br>SLE 2017	
29	Examination and observation	ANC_PROV_63	Inspected conjunctiva or examined the client for pallor	During the observed ANC visit, the provider inspected conjunctiva or examined the client for pallor.	Not collected in SARA; Collected only in some SPAs (those that include client/provider observation)	BGD 2014<br>SLE 2017	
30	Preventative treatment	ANC_PROV_43	Provided or prescribed iron pills and/or folic acid pills	During the observed ANC visit, the provider prescribed or provided iron pills or folic acid or both	Not collected in SARA; Collected only in some SPAs (those that include client/provider observation)	BGD 2014<br>SLE 2017	
31	Client education and counseling	ANC_PROV_65	Explained the purpose of iron or folic acid	During the observed ANC visit, the provider explained the purpose of iron or folic acid	Not collected in SARA; Collected only in some SPAs (those that include client/provider observation)	BGD 2014<br>SLE 2017	
32	Client education and counseling	ANC_PROV_66	Explained how to take iron or folic acid pills	During the observed ANC visit, the provider explained how to take iron or folic acid pills	Not collected in SARA; Collected only in some SPAs (those that include client/provider observation)	BGD 2014<br>SLE 2017	
33	Client education and counseling	ANC_PROV_68	Advised on potential side effects of iron or folic acid pills	During the observed ANC visit, the provider advised on potential side effects of iron or folic acid pills	Not collected in SARA; Collected only in some SPAs (those that include client/provider observation)	BGD 2014<br>SLE 2017	
34	Preventative treatment	ANC_PROV_80	Provided or prescribed albendazole or mebendazole	During the observed ANC visit, the provider prescribed or provided albendazole or mebendazole	Not collected in SARA; Collected only in some SPAs (those that include	BGD 2014<br>SLE 2017	

					client/provider observation)		
35	Client education and counseling	ANC_PROV_50a	Discussed exclusive breastfeeding	During the observed ANC visit, the provider discussed exclusive breastfeeding.	Not collected in SARA; Collected only in some SPAs (those that include client/provider observation)	BGD 2014<br>SLE 2017	
36	Client education and counseling	ANC_PROV_50b	Discussed early initiation and prolonged breastfeeding	During the observed ANC visit, the provider discussed early initiation and prolonged breastfeeding.	Not collected in SARA; Collected only in some SPAs (those that include client/provider observation)	BGD 2014<br>SLE 2017	
37	Client education and counseling	ANC_PROV_48	Discussed nutrition during the pregnancy	During the observed ANC visit, the provider discussed nutrition (i.e., quantity and quality of food to eat) during the pregnancy.	Not collected in SARA; Collected only in some SPAs (those that include client/provider observation)	BGD 2014<br>SLE 2017	
38	Preventative treatment	ANC_PROV_44	Provided or prescribed sulfadoxine-pyrimethamine	During the observed ANC visit, the provider prescribed or provided sulfadoxine-pyrimethamine	Not collected in SARA; Collected only in some SPAs (those that include client/provider observation; malaria endemic countries)	BGD 2014<br>SLE 2017	
39	Client experience	ANC_SATIS_03a	Client satisfied with the amount of explanation received about the problem or treatment	Client had no problems with regards to the amount of explanation she received about the problem or treatment.	Not collected in SARA; Collected only in some SPAs (those that include client/provider observation)	BGD 2014<br>SLE 2017	
40	Client experience	ANC_SATIS_10a	Client satisfied with how treated by the staff	Client had no problems with regards to how the staff treated her.	Not collected in SARA; Collected only in some SPAs (those that include client/provider observation)	BGD 2014<br>SLE 2017	
41	Client experience	ANC_SATIS_02a	Client satisfied with ability to discuss problems or concerns about pregnancy with provider	Client had no problems with regards to her ability to discuss the problems or concerns about her pregnancy.	Not collected in SARA; Collected only in some SPAs (those that include client/provider observation)	BGD 2014<br>SLE 2017	

Table S5: Overview of indicators and data sources used to define each step of the coverage cascade

Step of the coverage cascade	Service	Indicator(s)	Indicator abbreviation	Data source(s)
Target population	- ANC<br>- Maternal nutrition	Women ages 15-49 with a live birth in the last two years		Household survey
Service contact	- ANC<br>- Maternal nutrition	Proportion of women ages 15-49 with a live birth in the last two years who had at least 1 ANC contact during the most recent pregnancy	ANC1	Household survey
		Proportion of women ages 15-49 with a live birth in the last two years who had four or more ANC contacts during the most recent pregnancy	ANC4	
		Proportion of women ages 15-49 with a live birth in the last two years who had 8 or more ANC contacts during the most recent pregnancy	ANC8	
Readiness-adjusted coverage	- ANC	Proportion of women ages 15-49 with a live birth in the last two years who received ANC from an ANC "ready" facility<br><br>Readiness includes:<br>- ***Basic amenities:*** improved water source, room with auditory and visual privacy, sanitation facilities<br>- ***Diagnostics***: hemoglobin, urine dipstick- protein, urine dipstick- glucose, syphilis RDT / RPR, HIV diagnostic capacity<br>- ***Equipment and supplies***: blood pressure apparatus, stethoscope, examination bed, latex gloves, single use syringes with needles, handwashing materials, environmental disinfectant<br>- ***Human resources***: staff trained in the past 2 years in antenatal care<br>- ***Medicines and commodities***: iron tablets, folic acid tablets, tetanus toxoid vaccine		Household survey and Health facility assessment (facility inventory, Health worker interview)
	- Maternal nutrition	Proportion of women ages 15-49 with a live birth in the last two years who received ANC from a nutrition "ready" facility		

Step of the coverage cascade	Service	Indicator(s)	Indicator abbreviation	Data source(s)
		Readiness includes:<br>- ***Basic amenities***: clean environment, emergency transportation, improved water source, place for women to sit/lie down, power, sanitation facilities<br>- ***Diagnostics***: blood glucose, hemoglobin<br>- ***Equipment and supplies***: adult weighing scale, stadiometer or height rod, latex gloves, handwashing materials, environmental disinfectant, single use syringes with needles<br>- ***Human resources***: guidelines for antenatal care, guidelines for intermittent preventive treatment of malaria during pregnancy, guidelines for infant and young child feeding, staff trained in the past 2 years in antenatal care, staff trained in the past 2 years in ANC screening, staff trained in the past 2 years in counseling for ANC, staff trained in the past 2 years in nutritional assessment of the pregnant woman, staff trained in the past 2 years in complications of pregnancy and their management, staff trained in the past 2 years in infant and young child feeding<br>- ***Medicines and commodities***: albendazole/ mebendazole, folic acid tablets, iron tablets, sulfadoxine-pyrimethamine		
Intervention coverage	- ANC	Proportion of women ages 15-49 with a live birth in the last two years who reported receiving key interventions (average score of interventions received) during the most recent pregnancy<br><br>Interventions include:<br>- Tetanus toxoid vaccination[†]<br>o Received two doses of tetanus toxoid during this pregnancy OR at least 2 doses, the last within 3 years OR at least 3 doses, the last within 5 years OR at least 4 doses, the last within 10 years; OR at least 5 doses during lifetime	 <br><br><br><br>TT	Household survey

Step of the coverage cascade	Service	Indicator(s)	Indicator abbreviation	Data source(s)
		- SP for IPTp§<br>o At least 2 times during pregnancy<br>- Iron/folic acid supplementation<br>o Took at least 90 days of IFA during the pregnancy<br>- Blood pressure<br>o Measured at least once during the pregnancy<br>- Urine sample<br>o Taken at least once during the pregnancy<br>- Blood sample<br>o Taken at least once during the pregnancy<br>- Deworming<br>o Received drugs for intestinal parasites during pregnancy	SP for IPTp<br><br>IFA<br><br>BP	
	- Maternal nutrition	Proportion of women ages 15-49 with a live birth in the last two years who reported receiving key interventions (average score of interventions received) during the most recent pregnancy<br><br>Interventions include:		
		- SP for IPTp§<br>o At least 2 times during pregnancy	SP for IPTp	
		- Iron/folic acid supplementation<br>o Took at least 90 days of IFA during the pregnancy	IFA	
		- Blood sample<br>o Taken at least once during the pregnancy		
		- Deworming<br>o Received drugs for intestinal parasites during pregnancy		
Quality-adjusted coverage	- ANC	Proportion of women ages 15-49 with a live birth in the last two years who received ANC services according to standard protocols<br><br>Quality includes:		Household survey and Health facility assessment (ANC client

Step of the coverage cascade	Service	Indicator(s)	Indicator abbreviation	Data source(s)
		History taking: Provider asked about client age, Provider asked about medications client is taking, Provider asked about date last menstrual period began, Provider asked about any prior pregnancy, Provider asked about previous still birth, Provider asked about prior pregnancy in which the infant died in the first week of life, Provider asked about heavy bleeding during or after delivery in prior pregnancies, Provider asked about previous assisted delivery, Provider asked about previous spontaneous abortion, Provider asked about previous multiple pregnancies, Provider asked about previous prolonged labor, Provider asked about previous pregnancy-induced hypertension, Provider asked about previous pregnancy related convulsions, Provider asked about high fever or infection during prior pregnancy/ pregnancies, Provider asked about vaginal bleeding in current pregnancy, Provider asked about fever in current pregnancy, Provider asked about headache or blurred vision in current pregnancy, Provider asked about swollen face or hands or extremities in current pregnancy, Provider asked about tiredness or breathlessness in current pregnancy, Provider asked about fetal movement (loss of, excessive, normal) in current pregnancy, Provider asked about cough or difficulty breathing for 3 weeks or longer in current pregnancy<br>***Examination and observation:*** Observed client for oedema, Examined client blood pressure, Examined client weight, Palpated the clients' abdomen for fundal height<br>***Laboratory investigations:*** Performed or referred the client for hemoglobin testing, Performed or referred the client for grouping and rhesus factor testing, Performed or referred the client for syphilis testing, Performed or referred the client for HIV testing, Performed or referred the client for urine- protein, sugar, acetone testing		observation, ANC client exit interview)

Step of the coverage cascade	Service	Indicator(s)	Indicator abbreviation	Data source(s)
		Preventative treatment: Provided or prescribed iron pills and/or folic acid pills, Provided or prescribed tetanus toxoid immunization<br>***Client education and counseling:*** Discussed process of pregnancy and its complications, Discussed diet and nutrition, Discussed danger signs in pregnancy, Discussed voluntary counselling and testing for HIV, Discussed breastfeeding, Discussed plans of delivery<br>***Client experience:*** Client satisfied with ability to discuss problems or concerns about pregnancy with provider, Client satisfied with the amount of explanation received about the problem or treatment, Client satisfied with how treated by the staff, Client satisfied with privacy from having others see, Client satisfied with privacy from having others hear, Client satisfied with time waited, Client satisfied with number of days services are available, Client satisfied with the hours of service, Client satisfied with the cost for services or treatments, Client satisfied with the availability of medicines, Client satisfied with the cleanliness of the facility		
	- Maternal nutrition	Proportion of women ages 15-49 with a live birth in the last two years who received maternal nutrition services according to standard protocols<br><br>Quality includes:<br>***Examination and observation:*** Inspected conjunctiva or examined the client for pallor<br>***Laboratory investigations:*** Performed or referred the client for hemoglobin testing<br>***Preventative treatment:*** Provided or prescribed albendazole or mebendazole, Provided or prescribed sulfadoxine-pyrimethamine, Provided or prescribed iron pills and/or folic acid pills, Explained the purpose of iron or folic acid, Explained how to		

Step of the coverage cascade	Service	Indicator(s)	Indicator abbreviation	Data source(s)
		take iron or folic acid pills, Advised on potential side effects of iron or folic acid pills<br>***Client education and counseling:*** Discussed exclusive breastfeeding, Discussed early initiation and prolonged breastfeeding, Discussed nutrition during the pregnancy<br>***Client experience:*** Client satisfied with the amount of explanation received about the problem or treatment, Client satisfied with how treated by the staff, Client satisfied with ability to discuss problems or concerns about pregnancy with provider		

§ For SP for IPTp, the clinical guidelines were updated in 2013 followed by a change in the global monitoring indicator definition in 2016/2017 prompting us to use the older definition for pre-2016 surveys and the newer definition for post-2016 surveys [70,71]. However, the only post-2016 survey in our analysis did not ask about SP for IPTp thus effectively we only used the older SP for IPTp definition.

Ɨ For TT we used the most recent indicator definitions agreed upon by the international community [72,73].

Figure S1: Distribution of ANC readiness scores, by strata and country

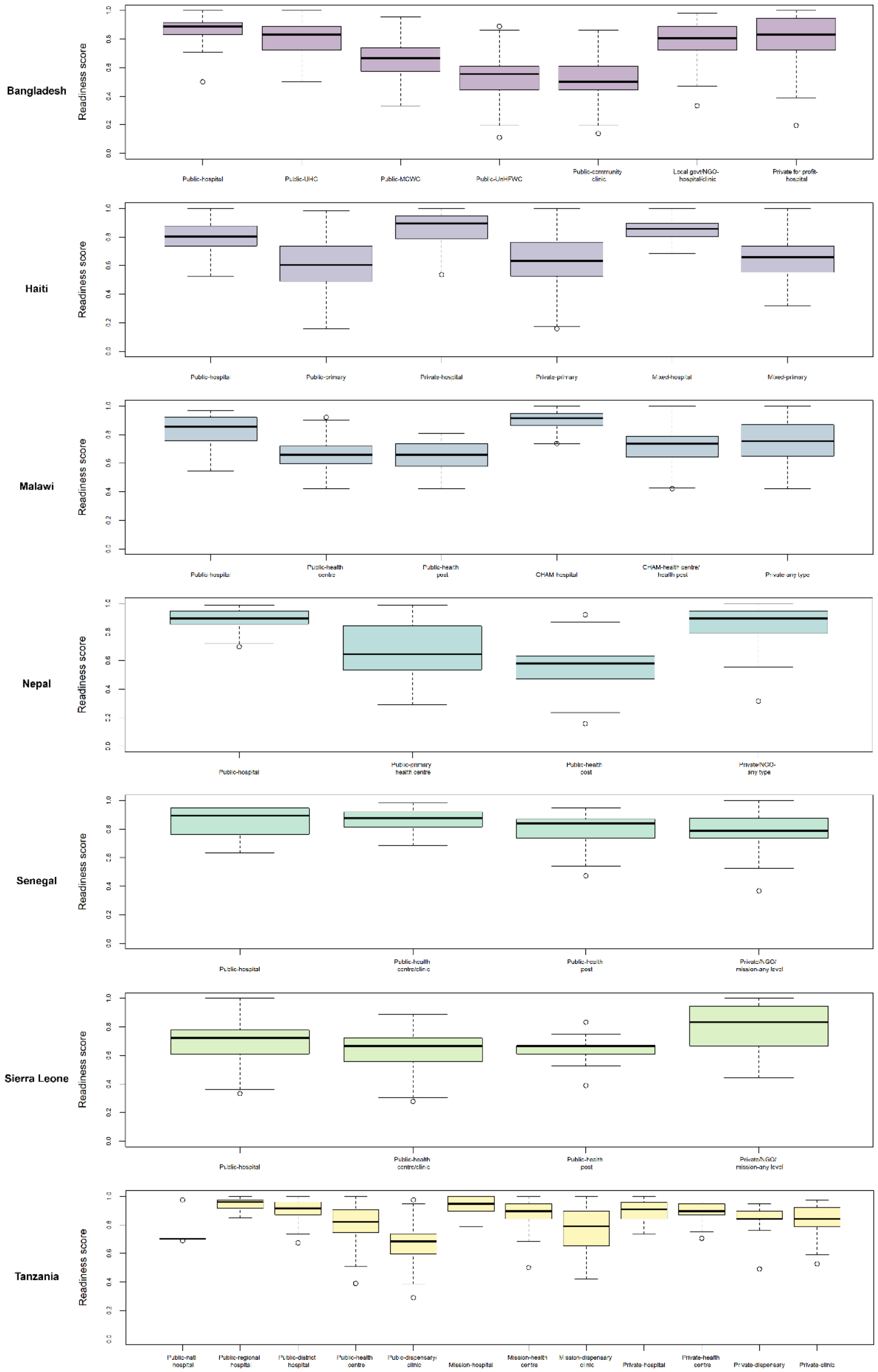

Figure S2: Distribution of maternal nutrition readiness scores, by strata and country

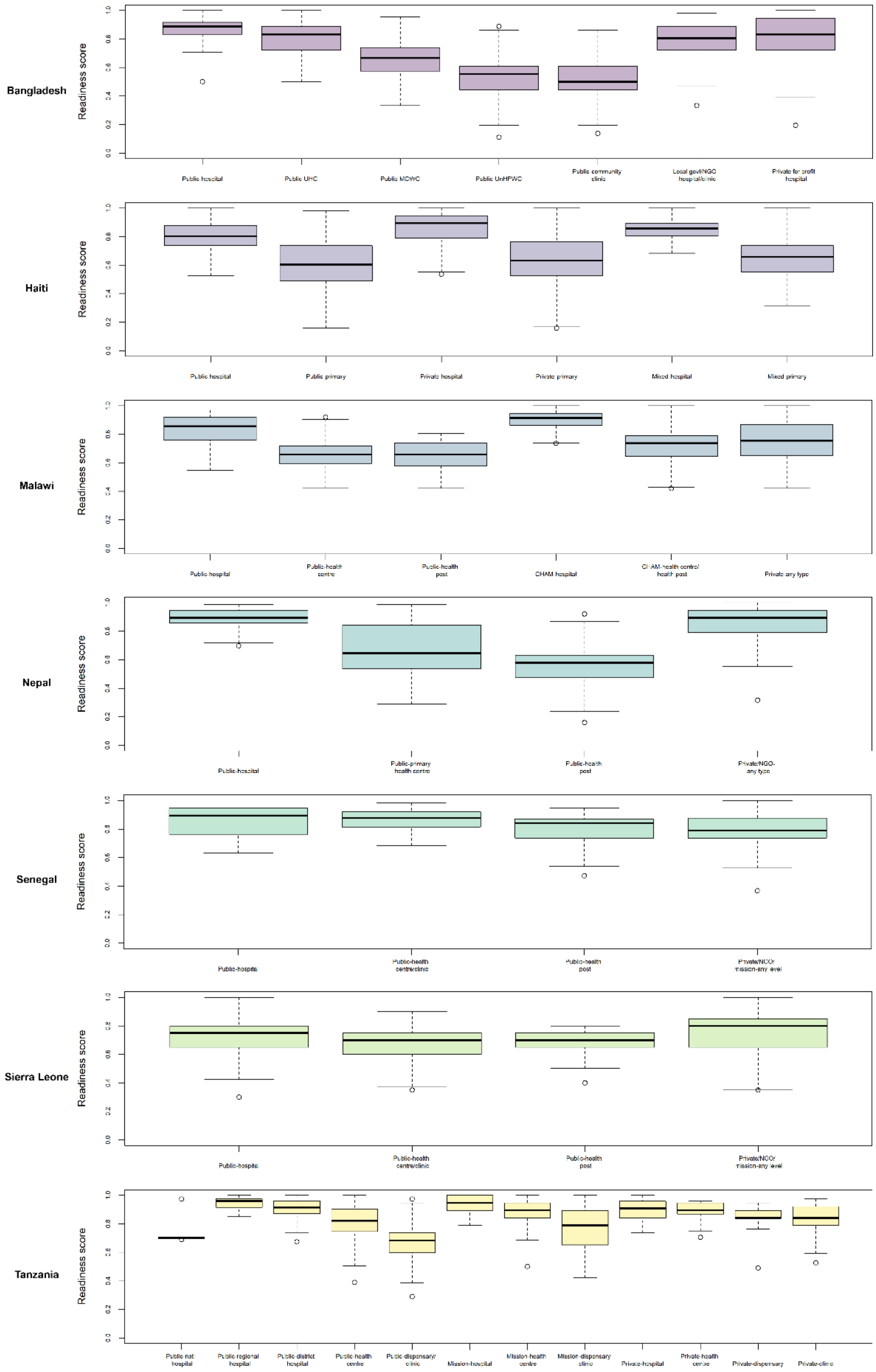

Table S6: Availability of ANC readiness items, by country

Item	Bangladesh N = 1493			Haiti N = 832			Malawi N = 643			Nepal N = 872			Senegal N = 323			Sierra Leone N = 1247			Tanzania N = 1032		
	Mean	CI_L	CI_U	Mean	CI_L	CI_U	Mean	CI_L	CI_U	Mean	CI_L	CI_U	Mean	CI_L	CI_U	Mean	CI_L	CI_U	Mean	CI_L	CI_U
Equipment																					
Blood pressure apparatus	87%	84%	90%	95%	93%	96%	68%	65%	72%	86%	83%	89%	98%	96%	100%	80%	78%	83%	79%	75%	83%
Stethoscope	92%	90%	94%	98%	97%	99%	75%	71%	78%	89%	86%	92%	94%	91%	97%	92%	91%	94%	83%	80%	87%
Examination bed	78%	74%	81%	91%	89%	93%	97%	96%	99%	92%	90%	95%	100%	100%	100%				98%	97%	100%
Latex gloves	64%	60%	68%	84%	82%	87%	96%	94%	97%	89%	86%	92%	99%	98%	100%	99%	98%	99%	90%	88%	93%
Single use syringes	82%	78%	85%	90%	88%	92%	98%	97%	99%	96%	94%	97%	80%	75%	85%	99%	98%	99%	95%	93%	97%
Soap and water OR alcohol-based hand rub	56%	52%	60%	73%	70%	76%	71%	68%	75%	58%	53%	63%	98%	96%	100%	91%	90%	93%	73%	70%	77%
Disinfectant	59%	55%	63%	82%	80%	85%	90%	88%	93%	80%	76%	84%	93%	90%	96%	87%	86%	89%	87%	84%	90%
Diagnostics																					
Hemoglobin	12%	10%	13%	33%	30%	36%	25%	22%	29%	14%	12%	16%	12%	10%	14%	4%	3%	5%	30%	26%	33%
Urine dipstick- protein	18%	16%	21%	39%	35%	42%	20%	17%	23%	16%	13%	18%	89%	85%	92%	22%	20%	24%	33%	29%	37%
Urine dipstick- glucose	18%	15%	21%	39%	35%	42%	18%	15%	21%	14%	12%	15%	86%	81%	90%	18%	16%	20%	29%	25%	32%
Syphilis RDT / RPR	6%	5%	6%	37%	34%	41%	29%	25%	32%	11%	10%	13%	4%	2%	6%	5%	3%	6%	52%	48%	57%
HIV testing / RDT				37%	33%	40%	95%	93%	96%	15%	13%	17%	91%	88%	95%	21%	16%	27%	90%	88%	93%
Medicines																					
Iron tablets	96%	94%	97%	82%	80%	85%	97%	96%	98%	92%	90%	94%	83%	79%	88%	61%	59%	95%	94%	93%	96%
Folic acid tablets	94%	92%	96%	75%	72%	78%	94%	92%	96%	92%	89%	94%	82%	77%	86%	89%	87%	79%	95%	94%	97%
TT vaccine	19%	16%	22%	35%	32%	39%	77%	74%	80%	25%	22%	29%	92%	89%	95%	88%	86%	83%	86%	83%	89%
Basic amenities and Human resources																					
Improved water source	87%	84%	90%	69%	66%	73%	95%	93%	96%	82%	78%	85%	95%	93%	98%	82%	80%	70%	65%	61%	69%
Room with auditory and visual privacy	29%	26%	33%	74%	71%	77%	78%	75%	82%	67%	63%	72%	59%	54%	65%	57%	54%	96%	73%	69%	76%
Sanitation facilities	87%	84%	90%	81%	78%	84%	90%	88%	93%	90%	87%	93%	100%	99%	100%	71%	68%	84%	66%	62%	70%
Proportion of staff offering ANC trained in ANC in last two years	16%	14%	19%	35%	33%	37%	37%	35%	40%	10%	9%	12%	68%	62%	73%	83%	81%	97%	34%	32%	37%
Overall score																					
Readiness score	55%	54%	56%	66%	65%	67%	71%	70%	72%	59%	58%	60%	80%	79%	81%	87%	85%	89%	71%	70%	72%

Table S7: Availability of maternal nutrition readiness items, by country

Item	Bangladesh N = 1493			Haiti N = 832			Malawi N = 643			Nepal N = 889			Senegal N = 323			Sierra Leone N = 1247			Tanzania N = 1031		
	Mean	CI_L	CI_U	Mean	CI_L	CI_U	Mean	CI_L	CI_U	Mean	CI_L	CI_U	Mean	CI_L	CI_U	Mean	CI_L	CI_U	Mean	CI_L	CI_U
Equipment																					
Adult weighing scale	83%	80%	86%	86%	83%	88%	88%	86%	91%	87%	83%	90%	97%	95%	99%	61%	58%	64%	84%	81%	87%
Stadiometer/height rod							60%	56%	64%										61%	57%	65%
Environmental disinfectant	59%	55%	63%	82%	80%	85%	90%	88%	93%	80%	76%	83%	93%	90%	96%	87%	86%	89%	87%	84%	90%
Single-use syringes with needles	82%	78%	85%	90%	88%	92%	98%	97%	99%	96%	94%	97%	80%	75%	85%	99%	98%	99%	95%	93%	97%
Handwashing materials	56%	52%	60%	73%	70%	76%	71%	68%	75%	58%	53%	62%	98%	96%	100%	91%	90%	93%	73%	70%	77%
Latex gloves	64%	60%	68%	84%	82%	87%	96%	94%	97%	89%	86%	92%	99%	98%	100%	99%	98%	99%	90%	88%	93%
Diagnostics																					
Hemoglobin	12%	10%	13%	33%	30%	36%	25%	22%	29%	14%	12%	15%	12%	10%	14%	4%	3%	5%	30%	26%	33%
Blood glucose	8%	6%	9%	37%	34%	41%	21%	18%	25%	7%	6%	8%	84%	80%	88%	5%	4%	7%	18%	15%	20%
Medicines																					
Iron tablets	62%	58%	66%	65%	62%	68%	37%	34%	41%	12%	10%	15%	6%	3%	8%	84%	82%	86%	41%	37%	45%
Folic acid tablets	94%	92%	96%	75%	72%	78%	94%	92%	96%	92%	89%	94%	82%	77%	86%	79%	77%	81%	95%	94%	97%
IPT drug (SP)							99%	98%	100%				75%	70%	80%	82%	80%	84%	61%	57%	65%
De-worming drugs (Mebendazole/Albendazole)	88%	85%	91%	88%	86%	90%	98%	96%	99%	97%	95%	98%	0%	0%	1%	88%	86%	90%	90%	88%	93%
Basic amenities																					
Place for women to sit/lie down (examination bed)	78%	74%	81%	91%	89%	93%	97%	96%	99%	92%	90%	95%	100%	100%	100%				98%	97%	100%
Power	21%	18%	24%	65%	61%	68%	67%	63%	70%	44%	40%	48%	58%	52%	64%	52%	49%	55%	64%	60%	69%
Improved water source	87%	84%	90%	69%	66%	73%	95%	93%	96%	81%	77%	85%	95%	93%	98%	57%	54%	59%	65%	61%	69%
Sanitation facilities	87%	84%	90%	81%	78%	84%	90%	88%	93%	90%	86%	93%	100%	99%	100%	83%	81%	85%	66%	62%	70%
Emergency transportation	9%	7%	10%	37%	34%	40%	83%	80%	86%	57%	52%	61%	64%	59%	70%	91%	90%	93%	59%	55%	64%
Clean environment	72%	70%	74%	91%	90%	92%	89%	88%	90%	82%	79%	84%	93%	92%	95%				82%	80%	84%
Human resources																					
Guidelines ANC	50%	46%	54%	33%	30%	37%	59%	55%	63%	25%	21%	29%	77%	72%	82%	62%	59%	65%	56%	51%	60%
Proportion of staff offering ANC trained in ANC in last two years	16%	14%	19%	35%	33%	37%	37%	35%	40%	10%	9%	12%	68%	62%	73%	87%	85%	89%	34%	32%	37%
Proportion of staff offering ANC trained in nutritional assessment of pregnant women in last two years	10%	8%	13%	13%	12%	15%	11%	9%	13%	5%	3%	7%	29%	25%	34%				6%	4%	7%
Guidelines IYCF				10%	8%	12%	30%	27%	34%				26%	21%	31%	38%	36%	41%	33%	29%	37%
Guidelines IPTP							49%	45%	53%				67%	61%	72%	64%	62%	67%	35%	31%	39%
Proportion of staff offering ANC trained in ANC screening in last two years	14%	11%	16%	14%	12%	16%	11%	9%	13%	8%	6%	10%	37%	32%	42%				8%	6%	10%
Proportion of staff offering ANC trained in counseling for ANC in last two years	16%	13%	19%	16%	14%	18%	13%	11%	15%	8%	6%	10%	38%	32%	43%				8%	6%	10%
Proportion of staff offering ANC trained in complications of pregnancy and their management in last two years	14%	12%	17%	15%	13%	17%	14%	11%	16%	8%	6%	11%	32%	27%	37%				8%	6%	10%
Proportion of staff offering ANC trained in infant and young child feeding in last two years	10%	7%	12%	10%	9%	12%	26%	23%	28%	3%	2%	4%	15%	12%	19%				27%	24%	30%
Overall score																					
Readiness score	47%	46%	48%	54%	53%	55%	61%	60%	62%	50%	49%	51%	63%	61%	64%	70%	70%	71%	55%	54%	56%

Figure S3: Distribution of ANC provision/experience of care scores, by strata and country

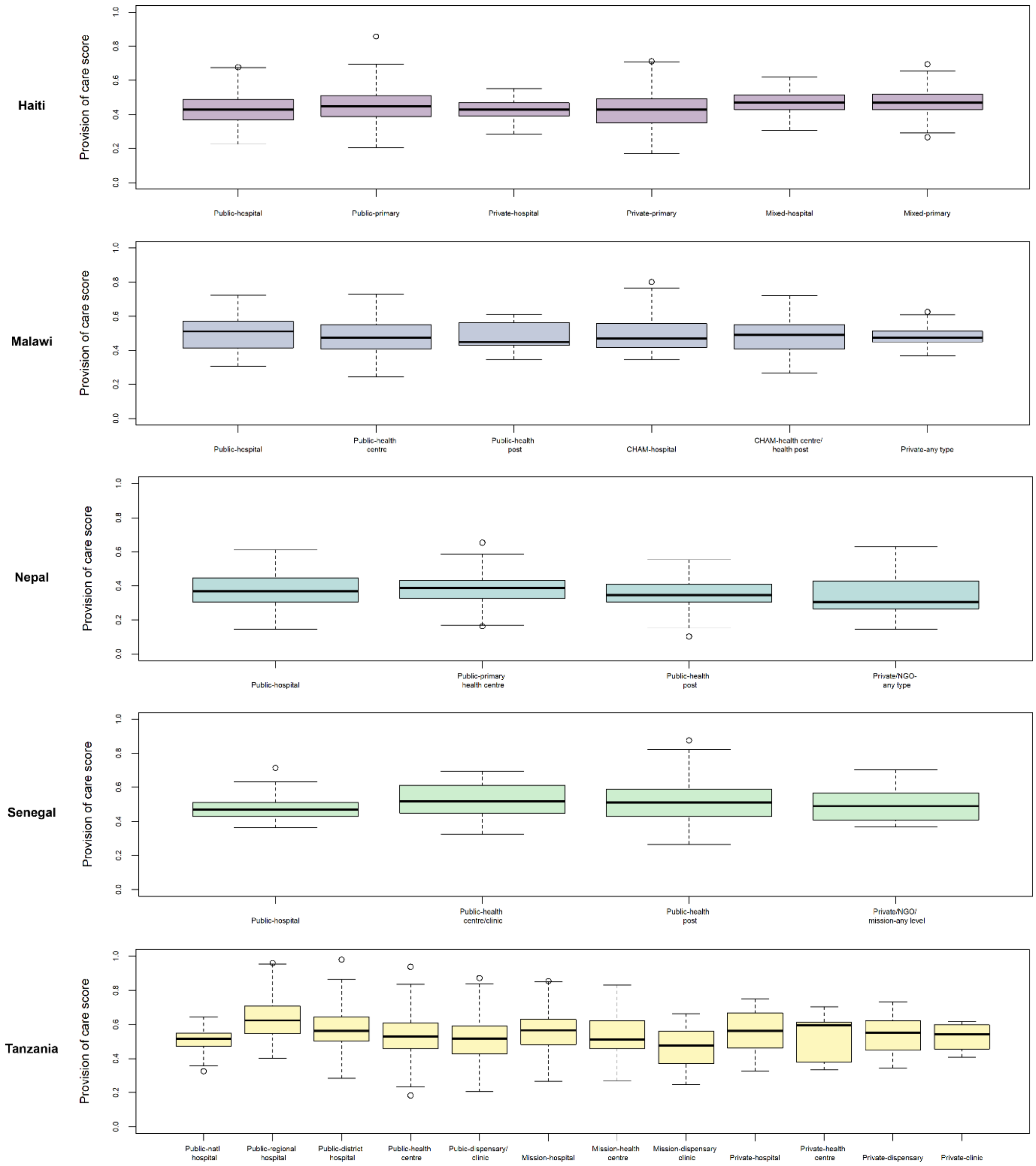

Figure S4: Distribution of maternal nutrition provision/experience of care scores, by strata and country

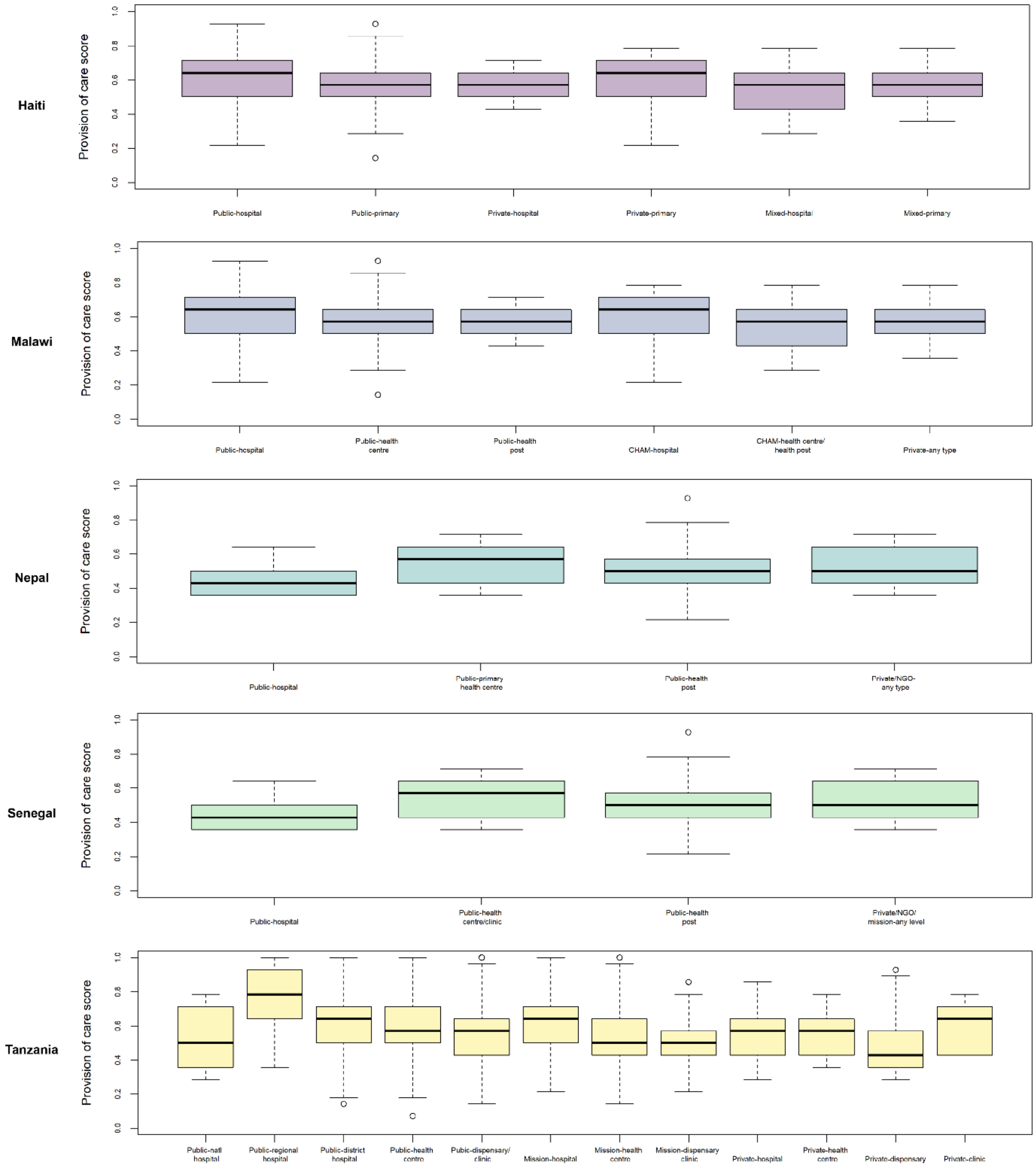

Table S8: Availability of ANC provision/experience of care items, by country

Item	Haiti N = 785			Malawi N = 859			Nepal N = 573			Senegal N = 307			Tanzania N = 1737		
	Mean	CI_L	CI_U	Mean	CI_L	CI_U	Mean	CI_L	CI_U	Mean	CI_L	CI_U	Mean	CI_L	CI_U
History taking: Personal and Past medical															
Personal history: client age	72%	69%	76%	80%	77%	83%	54%	48%	59%	91%	87%	95%	91%	89%	93%
Personal history: medications client is taking	11%	9%	13%	12%	9%	15%	12%	8%	15%	12%	8%	17%	12%	10%	14%
Personal history: date last menstrual period began	84%	81%	86%	76%	73%	79%	78%	72%	83%	73%	67%	79%	81%	78%	84%
Personal history: any prior pregnancy	73%	69%	76%	89%	86%	91%	63%	58%	69%	89%	85%	93%	87%	84%	89%
Past medical history for prior pregnancies: still birth	25%	22%	29%	43%	39%	47%	6%	4%	8%	48%	41%	54%	28%	25%	31%
Past medical history for prior pregnancies: infant died in the first week of life	9%	6%	11%	23%	20%	26%	1%	0%	2%	14%	9%	19%	24%	21%	26%
Past medical history for prior pregnancies: heavy bleeding during or after delivery	4%	2%	6%	37%	33%	41%	1%	0%	1%	4%	2%	7%	25%	22%	28%
Past medical history for prior pregnancies: previous assisted delivery	12%	9%	14%	47%	43%	51%	3%	2%	4%	6%	3%	9%	29%	26%	32%
Past medical history for prior pregnancies: previous spontaneous abortion	24%	20%	27%	46%	42%	50%	8%	5%	10%	20%	14%	25%	37%	34%	41%
Past medical history for prior pregnancies: previous multiple pregnancies	9%	7%	12%	22%	19%	25%	1%	0%	2%	63%	56%	69%	6%	4%	7%
Past medical history for prior pregnancies: previous prolonged labor	1%	0%	1%	8%	6%	10%	1%	0%	3%	21%	16%	27%	4%	3%	6%
Past medical history for prior pregnancies: pregnancy-induced hypertension	8%	6%	10%	29%	26%	33%	1%	0%	1%	3%	0%	5%	14%	12%	17%
Past medical history for prior pregnancies: pregnancy related convulsions	3%	1%	4%	26%	23%	30%	1%	0%	1%	15%	10%	19%	3%	2%	5%
Past medical history for prior pregnancies: high fever or infection during prior pregnancy	2%	1%	4%	3%	2%	4%	0%	0%	0%	4%	2%	7%	2%	1%	3%
History taking: Complaints in pregnancy															
History of complaints in current pregnancy: vaginal bleeding	14%	11%	16%	26%	23%	30%	9%	6%	11%	3%	1%	4%	50%	47%	53%
History of complaints in current pregnancy: fever	13%	11%	16%	11%	8%	13%	7%	4%	11%	3%	1%	5%	25%	22%	27%
History of complaints in current pregnancy: headache or blurred vision	31%	28%	35%	18%	16%	21%	14%	10%	18%	16%	11%	21%	42%	39%	45%
History of complaints in current pregnancy: swollen face or hands or extremities	5%	4%	7%	12%	10%	14%	19%	14%	24%	1%	0%	2%	31%	28%	34%
History of complaints in current pregnancy: tiredness or breathlessness	9%	7%	11%	5%	4%	7%	3%	2%	5%	6%	3%	9%	20%	18%	23%

History of complaints in current pregnancy: fetal movement (loss of, excessive, normal)	8%	6%	10%	22%	19%	25%	19%	15%	22%	11%	8%	15%	32%	29%	35%
History of complaints in current pregnancy: cough or difficulty breathing for 3 weeks or longer	7%	5%	9%	7%	5%	9%	1%	0%	1%	1%	0%	3%	5%	4%	7%
Observation and clinical investigation															
Edema	20%	17%	23%	65%	61%	68%	26%	21%	31%	86%	81%	91%	59%	55%	62%
Blood pressure	93%	91%	95%	48%	44%	52%	83%	79%	88%	99%	97%	100%	72%	69%	75%
Weight	81%	78%	84%	74%	70%	77%	72%	67%	78%	97%	94%	99%	79%	76%	82%
Palpate the client's abdomen for fundal height	57%	54%	61%	92%	91%	94%	17%	13%	20%	67%	61%	73%	79%	76%	81%
Laboratory investigation and Drug administration															
Hemoglobin	59%	56%	63%	10%	7%	12%	35%	29%	40%	40%	34%	47%	47%	44%	49%
Grouping and rhesus factor	37%	33%	41%	2%	1%	3%	32%	27%	37%	36%	30%	43%	34%	31%	36%
RPR (syphilis test)	53%	49%	56%	13%	10%	15%	19%	15%	22%	44%	37%	50%	42%	39%	45%
HIV testing	53%	49%	57%	53%	49%	56%	16%	12%	20%	65%	58%	71%	71%	68%	74%
Urine- protein, sugar, acetone	55%	52%	59%	4%	2%	6%	32%	27%	37%	65%	59%	71%	41%	39%	44%
Iron and/or folic acid provided or prescribed	73%	70%	77%	90%	88%	92%	71%	66%	77%	95%	92%	98%	83%	81%	86%
Tetanus toxoid provided or prescribed	25%	22%	28%	45%	41%	49%	31%	27%	36%	65%	59%	72%	52%	49%	55%
Client education and counselling															
Process of pregnancy and its complications	18%	15%	21%	57%	53%	60%	24%	19%	30%	23%	18%	29%	50%	47%	53%
Diet and nutrition	45%	41%	49%	44%	41%	48%	53%	47%	59%	43%	36%	49%	35%	31%	38%
Danger signs in pregnancy	6%	5%	7%	14%	12%	16%	2%	1%	3%	18%	14%	21%	36%	34%	37%
Voluntary counselling and testing for HIV	15%	12%	18%	41%	37%	45%	7%	5%	10%	64%	58%	71%	75%	72%	78%
Breastfeeding	7%	5%	9%	10%	7%	13%	2%	1%	4%	2%	1%	4%	16%	13%	18%
Plans of delivery (emergency preparedness, place of delivery, transportation, financial arrangements)	34%	30%	37%	82%	79%	85%	13%	9%	17%	20%	15%	26%	69%	66%	72%
Experience of care															
No problem: discuss problems or concerns	90%	88%	92%	93%	91%	95%	80%	75%	84%	99%	99%	100%	96%	95%	97%
No problem: explanation you received	92%	90%	94%	96%	94%	97%	78%	73%	82%	100%	100%	100%	96%	95%	97%
No problem: how the staff treated you	97%	96%	98%	94%	92%	96%	96%	94%	98%	100%	99%	100%	96%	95%	97%
No problem: privacy from having others see	93%	91%	95%	99%	98%	99%	84%	80%	89%	100%	100%	100%	96%	95%	97%
No problem: privacy from having others hear	92%	90%	94%	98%	97%	99%	85%	81%	89%	100%	100%	100%	96%	95%	97%
No problem: time you waited	72%	68%	75%	73%	70%	76%	66%	60%	71%	77%	72%	83%	69%	65%	72%
No problem: number of days services are available	93%	91%	95%	91%	89%	93%	90%	86%	94%	98%	97%	100%	92%	90%	94%
No problem: hours of service	94%	93%	96%	85%	83%	88%	91%	88%	94%	97%	96%	99%	90%	89%	92%
No problem: cost for services or treatments	93%	91%	95%	92%	90%	93%	92%	89%	95%	97%	95%	99%	94%	92%	95%
No problem: availability of medicines	90%	88%	92%	86%	83%	88%	87%	82%	91%	93%	90%	96%	78%	75%	80%
No problem: cleanliness of the facility	93%	92%	95%	92%	91%	94%	87%	84%	91%	98%	96%	100%	85%	83%	88%
Overall score															
Provision/experience of care score	44%	43%	45%	49%	48%	49%	36%	35%	37%	51%	50%	52%	53%	52%	53%

Table S9: Availability of nutrition provision/experience of care items, by country

Item	Haiti N = 785			Malawi N = 859			Nepal N = 573			Senegal N = 307			Tanzania N = 1754		
	Mean	CI_L	CI_U	Mean	CI_L	CI_U	Mean	CI_L	CI_U	Mean	CI_L	CI_U	Mean	CI_L	CI_U
Observation, laboratory investigation, and drug administration															
Hemoglobin	59%	56%	63%	10%	7%	12%	35%	29%	40%	40%	34%	47%	47%	44%	49%
Inspected conjunctiva or examined the client for pallor	52%	48%	56%	82%	79%	85%	34%	29%	40%	96%	94%	99%	62%	59%	65%
Antimalarials (SP) provided or prescribed				75%	71%	78%				34%	27%	40%	47%	44%	51%
Albendazole or mebendazole provided or prescribed	0%	0%	1%	71%	67%	74%	47%	41%	53%	18%	13%	23%	55%	52%	58%
Iron and/or folic acid provided or prescribed	73%	70%	77%	90%	88%	92%	71%	66%	77%	95%	92%	98%	83%	81%	86%
Provider gave counseling on purpose of iron tablets	28%	25%	32%	65%	61%	68%	26%	21%	32%	46%	40%	53%	65%	62%	68%
Provider gave counseling on how to take iron tablets	35%	31%	38%	64%	61%	68%	31%	25%	36%	49%	42%	55%	67%	64%	70%
Provider gave counseling on side effects of iron tablets	3%	1%	4%	10%	8%	12%	5%	3%	7%	3%	1%	4%	12%	10%	14%
Client education and counselling															
Diet and nutrition	45%	41%	49%	44%	41%	48%	53%	47%	59%	43%	36%	49%	35%	31%	38%
Exclusive breastfeeding	7%	5%	9%	10%	7%	12%	1%	0%	2%	2%	0%	3%	15%	12%	17%
Early initiation and prolonged breastfeeding	7%	5%	9%	10%	7%	12%	1%	0%	2%	1%	0%	3%	15%	12%	17%
Experience of care															
No problem: discuss problems or concerns	90%	88%	92%	93%	91%	95%	80%	75%	84%	99%	99%	100%	96%	95%	97%
No problem: explanation you received	92%	90%	94%	96%	94%	97%	78%	73%	82%	100%	100%	100%	96%	95%	97%
No problem: how the staff treated you	97%	96%	98%	94%	92%	96%	96%	94%	98%	100%	99%	100%	96%	95%	97%
Overall score															
Provision/experience of care score	45%	44%	46%	58%	57%	59%	43%	41%	45%	52%	50%	54%	57%	55%	58%

Figure S5: ANC8 effective coverage cascades, by country

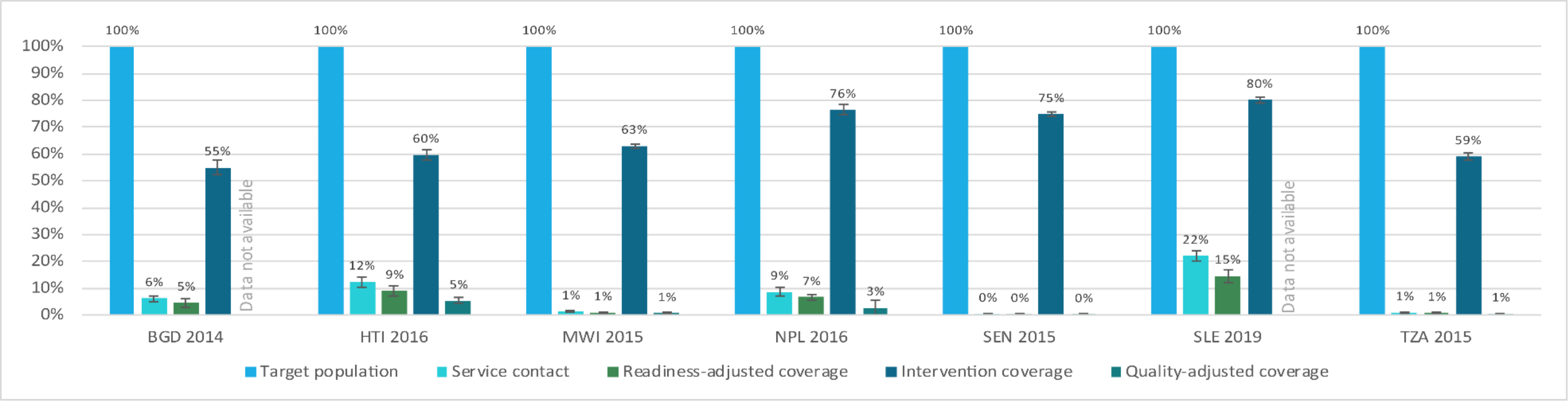

Table S10: ANC effective coverage cascade estimates, by country

	Target population	Service contact		Readiness-adjusted coverage		Intervention coverage		Quality-adjusted coverage	
	Women with a live birth in the last two years	Received at least 1/4/8 ANC visit(s)		Received ANC1/4/8 from a "ready" facility		Received ANC component interventions		Received ANC1/4/8 services according to standard protocols	
**ANC1**		**%**	**CI**	**%**	**CI**	**%**	**CI**	**%**	**CI**
BGD 2014	100%	80%	(77% - 83%)	59%	(55% - 63%)	55%	(52% - 58%)		
HTI 2016	100%	90%	(89% - 92%)	64%	(61% - 67%)	60%	(58% - 62%)	39%	(37% - 41%)
MWI 2015	100%	98%	(98% - 99%)	69%	(68% - 71%)	63%	(62% - 64%)	47%	(46% - 49%)
NPL 2016	100%	96%	(95% - 97%)	68%	(63% - 73%)	76%	(75% - 78%)	33%	(30% - 36%)
SEN 2015	100%	98%	(98% - 99%)	78%	(76% - 79%)	75%	(74% - 76%)	48%	(47% - 50%)
SLE 2019	100%	99%	(98% - 99%)	66%	(64% - 67%)	80%	(79% - 81%)		
TZA 2015	100%	98%	(97% - 98%)	72%	(70% - 74%)	59%	(58% - 60%)	51%	(50% - 53%)
**ANC4**									
BGD 2014	100%	31%	(29% - 34%)	23%	(19% - 26%)	55%	(52% - 58%)		
HTI 2016	100%	63%	(60% - 66%)	45%	(41% - 49%)	60%	(58% - 62%)	27%	(25% - 30%)
MWI 2015	100%	48%	(46% - 50%)	34%	(33% - 36%)	63%	(62% - 64%)	23%	(22% - 24%)
NPL 2016	100%	71%	(68% - 74%)	51%	(47% - 54%)	76%	(75% - 78%)	24%	(19% - 29%)
SEN 2015	100%	56%	(53% - 58%)	44%	(42% - 47%)	75%	(74% - 76%)	27%	(26% - 29%)
SLE 2019	100%	79%	(78% - 81%)	53%	(50% - 56%)	80%	(79% - 81%)		
TZA 2015	100%	48%	(46% - 50%)	36%	(33% - 39%)	59%	(58% - 60%)	26%	(23% - 28%)
**ANC8**									
BGD 2014	100%	6%	(5% - 7%)	5%	(3% - 6%)	55%	(52% - 58%)		
HTI 2016	100%	12%	(11% - 14%)	9%	(7% - 11%)	60%	(58% - 62%)	5%	(4% - 6%)
MWI 2015	100%	1%	(1% - 2%)	1%	(1% - 1%)	63%	(62% - 64%)	1%	(0% - 1%)
NPL 2016	100%	9%	(7% - 10%)	7%	(6% - 8%)	76%	(75% - 78%)	3%	(0% - 5%)
SEN 2015	100%	0%	(0% - 1%)	0%	(0% - 1%)	75%	(74% - 76%)	0%	(0% - 0%)
SLE 2019	100%	22%	(20% - 24%)	15%	(12% - 17%)	80%	(79% - 81%)		
TZA 2015	100%	1%	(1% - 1%)	1%	(0% - 1%)	59%	(58% - 60%)	1%	(0% - 1%)

Figure S6: ANC8 maternal nutrition effective coverage cascades, by country

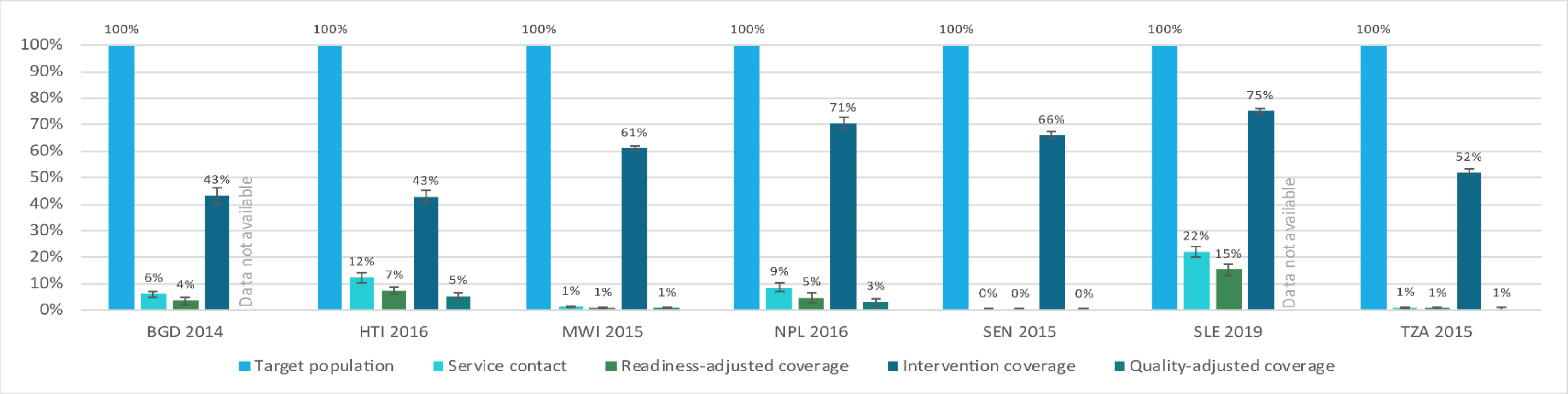

Table S11: Maternal nutrition effective coverage cascade estimates, by country

	Target population	Service contact		Readiness-adjusted coverage		Intervention coverage		Quality-adjusted coverage	
	Women with a live birth in the last two years	Received at least 1/4/8 ANC visit(s)		Received ANC1/4/8 from a nutrition "ready" facility		Received nutrition component interventions		Received ANC1/4/8 services according to standard protocols for maternal nutrition	
**ANC1**		%	CI	%	CI	%	CI	%	CI
BGD 2014	100%	80%	(77% - 83%)	47%	(43% - 50%)	43%	(40% - 46%)		
HTI 2016	100%	90%	(89% - 92%)	52%	(50% - 55%)	43%	(41% - 45%)	40%	(38% - 42%)
MWI 2015	100%	98%	(98% - 99%)	60%	(59% - 62%)	61%	(60% - 62%)	56%	(54% - 58%)
NPL 2016	100%	96%	(95% - 97%)	51%	(49% - 54%)	71%	(68% - 73%)	40%	(34% - 45%)
SEN 2015	100%	98%	(98% - 99%)	62%	(60% - 64%)	66%	(65% - 67%)	50%	(46% - 53%)
SLE 2019	100%	99%	(98% - 99%)	69%	(67% - 70%)	75%	(74% - 76%)		
TZA 2015	100%	98%	(97% - 98%)	55%	(53% - 56%)	52%	(51% - 53%)	56%	(54% - 58%)
**ANC4**									
BGD 2014	100%	31%	(29% - 34%)	18%	(15% - 21%)	43%	(40% - 46%)		
HTI 2016	100%	63%	(60% - 66%)	37%	(34% - 40%)	43%	(41% - 45%)	28%	(26% - 30%)
MWI 2015	100%	48%	(46% - 50%)	30%	(28% - 31%)	61%	(60% - 62%)	27%	(26% - 29%)
NPL 2016	100%	71%	(68% - 74%)	38%	(35% - 42%)	71%	(68% - 73%)	29%	(24% - 34%)
SEN 2015	100%	56%	(53% - 58%)	35%	(33% - 37%)	66%	(65% - 67%)	28%	(26% - 31%)
SLE 2019	100%	79%	(78% - 81%)	56%	(53% - 58%)	75%	(74% - 76%)		
TZA 2015	100%	48%	(46% - 50%)	28%	(25% - 30%)	52%	(51% - 53%)	28%	(25% - 30%)
**ANC8**									
BGD 2014	100%	6%	(5% - 7%)	4%	(2% - 5%)	43%	(40% - 46%)		
HTI 2016	100%	12%	(11% - 14%)	7%	(6% - 9%)	43%	(41% - 45%)	5%	(4% - 7%)
MWI 2015	100%	1%	(1% - 2%)	1%	(1% - 1%)	61%	(60% - 62%)	1%	(0% - 1%)
NPL 2016	100%	9%	(7% - 10%)	5%	(3% - 7%)	71%	(68% - 73%)	3%	(2% - 4%)
SEN 2015	100%	0%	(0% - 1%)	0%	(0% - 0%)	66%	(65% - 67%)	0%	(0% - 0%)
SLE 2019	100%	22%	(20% - 24%)	15%	(13% - 18%)	75%	(74% - 76%)		
TZA 2015	100%	1%	(1% - 1%)	1%	(0% - 1%)	52%	(51% - 53%)	1%	(0% - 1%)

Figure S7: Comparing ANC and maternal nutrition readiness-adjusted and quality-adjusted coverage, by country

A) ANC1

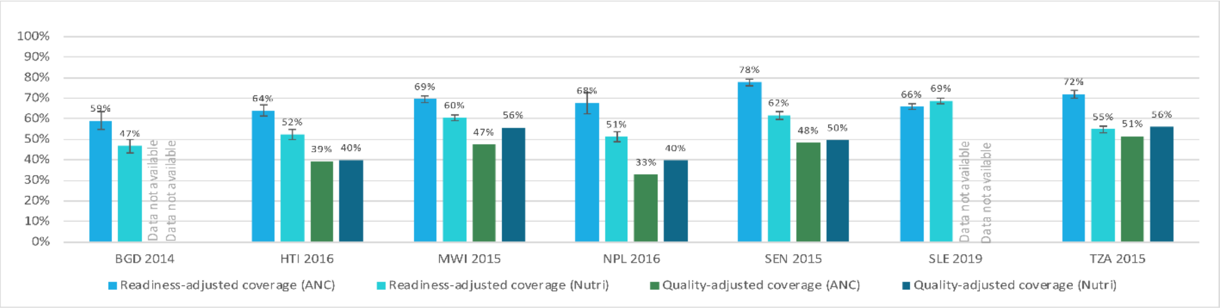

B) ANC4

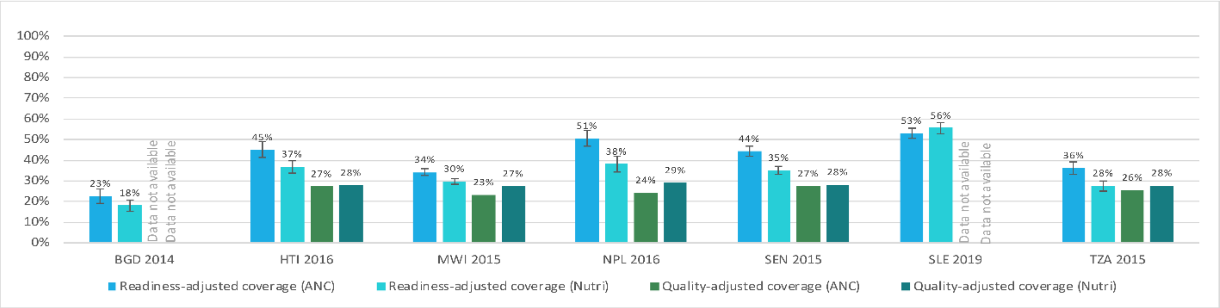

C) ANC8

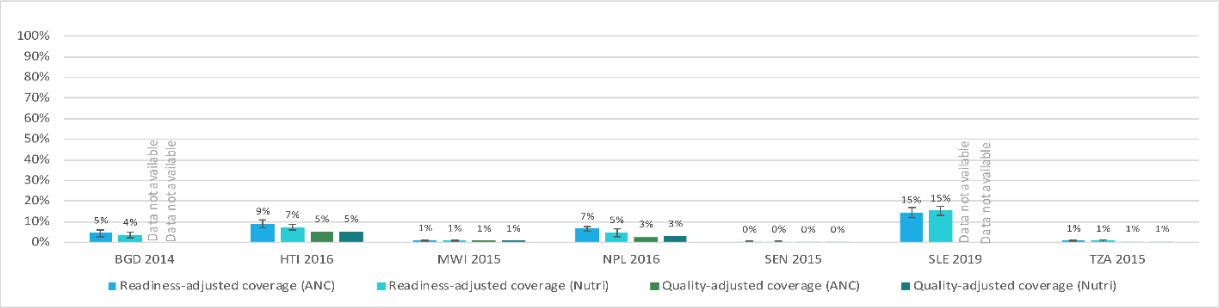

Supplement: Online Supplementary Document [file jogh-15-04041-s001.pdf]
